# Unsupervised ranking of clustering algorithms by INFOMAX

**DOI:** 10.1371/journal.pone.0239331

**Published:** 2020-10-26

**Authors:** Sandipan Sikdar, Animesh Mukherjee, Matteo Marsili

**Affiliations:** 1 RWTH Aachen University, Aachen, Germany; 2 Indian Institute of Technology Kharagpur, Kharagpur, India; 3 Abdus Salam International Centre for Theoretical Physics, Trieste, Italy; University of Bradford, UNITED KINGDOM

## Abstract

Clustering and community detection provide a concise way of extracting meaningful information from large datasets. An ever growing plethora of data clustering and community detection algorithms have been proposed. In this paper, we address the question of *ranking* the performance of clustering algorithms for a given dataset. We show that, for hard clustering and community detection, Linsker’s Infomax principle can be used to rank clustering algorithms. In brief, the algorithm that yields the highest value of the entropy of the partition, for a given number of clusters, is the best one. We show indeed, on a wide range of datasets of various sizes and topological structures, that the ranking provided by the entropy of the partition over a variety of partitioning algorithms is strongly correlated with the overlap with a ground truth partition The codes related to the project are available in https://github.com/Sandipan99/Ranking_cluster_algorithms.

## 1 Introduction

Cluster analysis is being increasingly used across wide range of applications ranging from biology and bioinformatics [1] to social networks [2] which has led to the development of a plethora of clustering algorithms. Given this, an obvious query that arises is how do we evaluate the performance of these algorithms in terms of the clusters obtained from them. In this paper, we show evidence in support of the idea that the Infomax principle [3] provides an answer to this question.

**Clustering problem**: We focus on the problem of hard partitioning: given a list of objects (or data points) the problem is that of dividing them into groups of similar ones. In the computer science and pattern recognition literature, this problem is popularly known as clustering. A plethora of different algorithms have been proposed for clustering (see [4, 5] for reviews) based on different measures of similarity between the data points. A large part of this literature has focused on the time complexity of the methods, which is particularly relevant for big data.

**Quality of clusters**: In this paper, we focus on the *quality*, i.e., on the accuracy of the method in terms of the results produced. Several algorithms (see e.g. [6, 7]) have been proposed claiming superior performance, yet it has been proven that no single clustering algorithm simultaneously satisfies a set of basic desiderata of data clustering [8]. In addition, the criteria for assessing the quality or validity of a clustering structure is not unique [4, 5]. When no ground truth is available, which is typically the case, (*internal*) criteria have been proposed based on stability [9] or on generalisability with respect to sub-sampling [10]. When a ground truth is available, an *external* criteria is possible, based on the distance of the predicted clustering to the ground truth. Yet the choice of the distance measure used is not unique [5]. Even in cases where comparison with a ground truth is possible, different algorithms are found to perform better in different cases and the predicted structures may differ substantially from the ground truth [11].

**Infomax principle for measuring quality**: We primarily intend to show that the Infomax principle [3] provides a natural measure for ranking clustering algorithms, for a given dataset, with respect to an *unknown* ground truth. In brief, a clustering algorithm is a mapping between data points *x*_*i*_ in a high dimensional feature space to a set of labels *s*_*i*_. The amount of information that the cluster structure retains about the data is given by the mutual information *I*(*x*, *s*) = *H*[*s*] − *H*[*s*|*x*]. The Infomax principle states that the optimal representation is the one that maximizes *I*(*s*, *x*). In hard clustering *H*[*s*|*x*] = 0, so *I*(*x*, *s*) = *H*[*s*] coincides with the entropy of the labels. We can visualize clustering as a translation of a dataset into a set of symbols—the cluster labels—of an alphabet of *S* letters, where *S* is the number of clusters. So, each partitioning algorithm is a *translator* that converts high dimensional data to a message. Following Shannon [12], the entropy $\hat{H}\left[s\right]$ of the cluster labels *s* provides a natural measure of the amount of information that the algorithm extracts from the data. Infomax then prescribes that the algorithm that “uses the most informative language”—i.e., with the highest $\hat{H}\left[s\right]$—should be preferred. *This allows one to rank partitioning algorithms in a completely unsupervised fashion, for a given dataset*—the fundamental contribution of this paper. So, this criterion is *internal*, in the sense that it is based only on the data (i.e., it is unsupervised), but we will validate it showing that the obtained ranking has a positive correlation with distance to a ground truth in all of the cases analyzed, and this correlation is strong in most cases. Our results are based on an extensive comparison across different algorithms, different similarity metrics and different databases for data clustering.

**Contributions**: Our contributions in this paper are threefold -

1. We propose a metric ($\hat{H}\left[s\right]$) which is able to rank, very efficiently, the clustering algorithms in a completely unsupervised way (i.e., without considering the ground truth cluster structure).
2. Through rigorous experiments across a wide range of datasets we show the effectiveness of our metric in ranking the performance of data clustering algorithms. In fact, the metric remarkably correlates with the distance from the ground truth for a widely *varying taxonomies of ground truth structures* including (i) ground truth with different granularities, (ii) ground truth built from different attributes, (iii) very small number of ground truth clusters, (iv) ground truth clusters with very few data points, (v) ground truth clusters of equal sizes and (vi) ground truth clusters with skewed sizes.
3. The proposed metric also outperforms the existing unsupervised metrics across all the datasets.

## 2 Background

In this section we present a brief overview of the related literature encompassing clustering algorithms and cluster quality measurement metrics used in our work.

### 2.1 Clustering algorithms

We consider two broad classes of clustering algorithms (i) hierarchical and (ii) partitional.

**Hierarchical methods**: These methods construct clusters through recursive partitioning of the data points in a bottom-up approach whereby each data point is assigned a cluster of its own initially and is merged until the desired number of clusters are obtained. The merging of the clusters is obtained according to some chosen similarity measure. We consider both city-block (l1) and Euclidean (l2) distance based similarity measures. The hierarchical clustering methods can be further classified according to the manner in which the similarity measure is calculated. We consider the following three classical ways—(1) **Single linkage (SI)** [13], (2) **Complete linkage (CO)** [14] and (3) **Average linkage (AV)** [15] Note that ‘l1SI’ would mean single linkage with city-block as distance metric and so on. We use this combination of acronyms for the algorithms and distance metrics in all our results presented in the subsequent sections.

We also consider **BIRCH (BI)** (balanced iterative reducing and clustering using hierarchies) [16] which improves upon the traditional hierarchical clustering methods. The algorithm commences by creating a height balanced tree out of the data points followed by execution of an agglomerative clustering method to obtain sub clusters.

**Partitional methods**: Among partitional methods we consider **K-means, affinity propagation** and **spectral clustering**. **k-means (KM)** clustering method which employs a squared error minimization criteria and is the most commonly used clustering technique in this category. The algorithm starts with an initial set of clusters chosen at random. In each round, each instance is assigned to its nearest cluster center according to distance between the two (we consider both l1 and l2 distances).

**Affinity propagation (AP)** algorithm introduced in [7] is based on the concept of passing messages between the data points. Unlike *k*-means clustering which identifies an exemplar (centroid) for each cluster, AP considers every data point to be a possible exemplar, representing a cluster. The goal is to obtain an appropriate set of exemplars which represents all the clusters.

**Spectral clustering (SI)** [17] employs a low dimensional embedding of the similarity matrix between the data points which is followed by clustering of eigenvector components in the low dimensional space.

### 2.2 Quality of cluster structure

The metrics available for determining the quality of clusters and thereby evaluating the performance of the clustering algorithms can be categorized as (i) external or supervised, which utilizes a benchmark or a ground truth cluster structure to determine quality and (ii) internal or unsupervised, which takes into account only the similarity between the data points used for clustering.

**External metrics**. Most commonly used external metrics are (i) **purity** [18], (ii) **normalized mutual information (NMI)** [19] and (iii) **adjusted rand index (ARI)** [20]. We explain them below.

Let Ω = (*ω*_1_, *ω*_2_, …, *ω*_*K*_) represent the set of clusters, $C=(c_{1},c_{2},\ldots ,c_{J})$ denote the set of ground truth classes and *N*, the number of data points.

- **Purity**: Purity value between Ω and C is calculated as -
  $$\begin{array}{c} Purity\left(\Omega ,C\right)=\frac{1}{N}\sum_{k}\underset{j}{\text{max}}\left(\omega_{k}\cap c_{j}\right) \end{array}$$ (1)
- **Normalized mutual information (NMI)**: NMI value between Ω and C is calculated as -
  $$\begin{array}{c} NMI\left(\Omega ,C\right)=\frac{2*I(\Omega ,C)}{H(\Omega )+H(C)} \end{array}$$ (2)
  where I is the mutual information and is defined as -
  $$\begin{array}{c} I\left(\Omega ,C\right)=\sum_{k}\sum_{j}\frac{|\omega_{k}\cap c_{j}|}{N}\log\frac{N|\omega_{k}\cap c_{j}|}{|\omega_{k}\left|\right|c_{j}|} \end{array}$$ (3)
  and H is the entropy.
- **Adjusted rand index (ARI)**: ARI is a corrected version of rand index and its value between Ω and C is calculated as -
  ARI(Ω,C)=∑kj(nkj2)-[∑k(ak2)∑j(bj2)]/(N2)12[∑k(ak2)+∑j(bj2)]-[∑k(ak2)∑j(bj2)]/(N2) (4)
  where *n*_*kj*_ = |*ω*_*j*_ ∩ *c*_*k*_|, *a*_*k*_ is the size of *ω*_*k*_ and *b*_*j*_ is the size of *c*_*j*_. Other measures include Jaccard index [21], Dice index [22] and Fowlkwes-Mallows index [23].

**Internal metrics**. Internal metrics for evaluation include Davies-Bouldin index [24], Silhouette [25] and Dunn index [26]. Among these we compare our proposed metric with Davies-Bouldin index **DB** and Silhouette **SH**. DB can be calculated as

$$\begin{array}{c} DB=\frac{1}{n}\sum_{i-1}^{n}max_{j\neq i}\frac{\sigma_{i}+\sigma_{j}}{d(c_{i},c_{j})} \end{array}$$ (5)

where *n* is the number of clusters, *c*_*x*_ is the centroid of cluster *x*, *σ*_*x*_ represents the average distance of all elements in cluster *x* to centroid *c*_*x*_ and *d*(*c*_*i*_, *c*_*j*_) is the distance between centroids *c*_*i*_ and *c*_*j*_.

For each data point, SH is computed utilizing the mean intra-cluster distance *a*, and its distance from the nearest cluster that it is not a part of *b*, with the score obtained as $\frac{\left(b-a\right)}{max(a,b)}$. The overall score is computed as the mean over all the individual data points.

## 3 Proposed metric

In this section we first discuss the clustering problem and then introduce our proposed metric which ranks the clustering and community detection algorithms in a completely unsupervised way.

### 3.1 Clustering problem

Consider a dataset composed of *M* points $x\in R^{d}$ in a high dimensional feature space (*d* ≫ 1). The primary objective of clustering is to assign each point *x*_*i*_ a label *s*_*i*_ that indicates the partition to which point *x*_*i*_ belongs to. If there are *S* partitions, *s*_*i*_ can be taken as an integer between 1 and *S*. A data clustering algorithm [4, 5] partitions objects *x*_*i*_ into groups or clusters of “similar” objects, where similarity is defined in terms of a metric distance.

With numerous clustering algorithms available for this specific task and ground truth not always available, we in this paper intend to propose a metric which ranks these algorithms based on their performance in a completely unsupervised way (i.e., without considering ground truth partition).

### 3.2 Infomax based metric $\hat{H}\left[s\right]$

For a given data set and number of clusters *S*, each algorithm assigns to each point *x*_*i*_ in the sample a label *s*_*i*_ in an alphabet of *S* possible labels. Loosely speaking, each algorithm *translates* the data into a message of a language written in this alphabet. The information content of this message can be quantified by the Shannon entropy. Assuming the order in which the data occur to be uninformative, as is often the case, the information is stored uniquely in the symbol frequencies, i.e. in the number *K*_*s*_ of times that a symbol *s* occurs (which is the size of cluster *s*). As an estimate of the amounts of bit of information per character in the message we take

$$\begin{array}{c} \hat{H}\left[s\right]=-\sum_{s}\frac{K_{s}}{M}\log\frac{K_{s}}{M}. \end{array}$$ (6)

**Fig 1:**
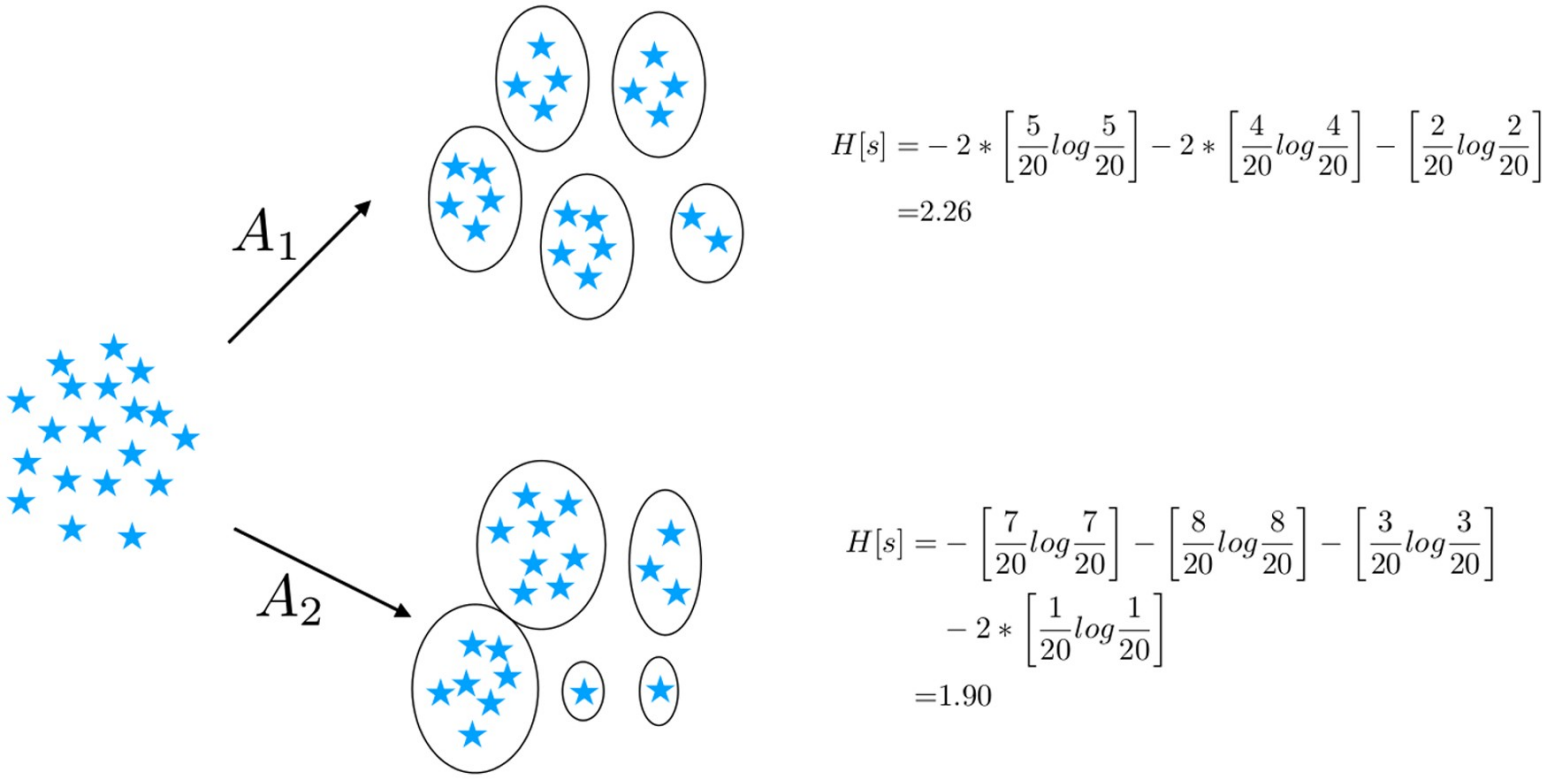
In this example there are 20 points that need to be clustered. The number of clusters is set at 5 and we deploy two algorithms *A*_1_ and *A*_2_ which generate clusters of sizes {5, 5, 4, 4, 2} and {7, 8, 3, 1, 1} respectively. Our metric assigns a higher score to the cluster output of *A*_1_ (2.26) and thus inferring it to be better than *A*_2_.

The Infomax principle [27] suggests a natural and universal criterium for scoring different algorithms: If algorithm *A*_1_ extracts more information than *A*_2_ from a dataset, i.e. if ${\hat{H}}_{A_{1}}\left[s\right]>{\hat{H}}_{A_{2}}\left[s\right]$, then *A*_1_ should be preferred. For a given dataset and a fixed *S*, $\hat{H}\left[s\right]$ can be measured on the cluster predicted by different algorithms, thereby providing an un-supervised ranking of the algorithms. To summarize, given a cluster output of an algorithm consisting of *S* clusters, our metric essentially quantifies the quality of the cluster output by computing the entropy of the cluster labels. We illustrate using a toy example in Fig 1.

### 3.3 Advantages

The proposed metric has several advantages which we summarize below -

- **Model-free**. The proposed metric is model-free which allows for its application across any clustering algorithm and dataset.
- **Information theory-based**. Unlike the existing internal metrics, our metric builds upon information theory which is already deep-rooted in the existing literature making our metric much more reliable.
- **Outperforms existing metrics**. Our metric consistently outperforms the existing internal metrics across numerous datasets (refer to section 6 for details).
- **Unsupervised**. In contrast to the existing external metrics, our metric does not require ground truth cluster structure making it completely unsupervised and hence suited to a wide range of datasets. Even though it requires less information, the proposed metric provides comparable performance to the external metrics (refer to section 6 for details).

## 4 Datasets

In this section we briefly discuss the datasets that we have used in this paper.

**Abalone**: The Abalone dataset https://archive.ics.uci.edu/ml/datasets/Abalone consists of a set of abalone and are classified based on their age which is basically the number of rings they have [28]. The dataset consists of 4177 instances each consisting of 8 attributes. The task is treated as a classification problem and there are 28 clusters in the ground truth.

**Football**: The Football network [29] http://www-personal.umich.edu/mejn/netdata/ consists of American football games between Division IA colleges during regular season Fall of 2000. The vertices in the network are the football teams which are identified by the respective college names and an edge in the network represent regular season games between the two teams. The teams are divided into conferences containing around 8–12 teams each. Games are more frequent between members of the same conference than between members of different conferences. Each conference therefore represents a ground truth community in the network. Note the vertices in the network are devoid of any inherent features and we hence resort to representing each vertex by vectors of (i) neighborhood (1 if the corresponding vertex is a neighbor and 0 other) and (ii) shortest path (length of shortest path to the corresponding vertex).

**Railway**: The Indian railway network was proposed in [30] http://www.cnergres.iitkgp.ac.in/permanence/ and it consists of stations (nodes) and edges between all pairs of stations that are connected by at least one train-route (both stations must be scheduled halts on the train-route). The weight of the edge between two stations is the number of train-routes on which both these stations are scheduled halts. We filter out the low-weight edges and then make the resultant network unweighted. The states act as communities since the number of trains within each state is much higher than the number of trains in between two states. Similar to the Football dataset we again obtain two representations of each vertex (neighborhood and shortest path).

**Wine**: We consider two wine datasets namely Red and White wine [31] http://archive.ics.uci.edu/ml/datasets/Wine+Quality. The datasets respectively contain samples of red and white wines. Each wine sample is associated with 11 attributes like fixed acidity, volatility, residual sugar etc. Each wine sample is also graded by experts between 0 (very bad) and 10 (very excellent) based on the quality. This quality score acts as the ground truth cluster for the two datasets.

**Leaf**: The leaf dataset [32] https://archive.ics.uci.edu/ml/datasets/One-hundred+plant+species+leaves+data+set consists of 100 varieties of leaves and for each variety there are 16 examples. Each leaf sample is associated with a shape, texture and margin feature. Each such feature is a vector of 64 elements. Each variety of leaf act as the ground truth cluster.

**TREC**: The TREC dataset [33] http://glaros.dtc.umn.edu/gkhome/views/cluto consists of articles from the Los Angeles times and the categories correspond to the desk of the paper that each article appeared and include documents from the entertainment, financial, foreign, metro, national, and sports desks. Frequency of words in the document are its associated features. A stop-list was used to remove the common words and any word occurring in less than two documents was eliminated. Each desk here represents a ground truth cluster.

**Synthetic**: The dataset is obtained using the model of correlated time series discussed in [34]. The dataset consists of 1000 data points and 68 clusters in the ground-truth. The dataset https://www.kaggle.com/sandipan99/synthetic-data-for-clustering has been made public.

**Protein**: This dataset http://www.fludb.org/brc/home.spg?decorator=influenza consists of sequences of HA1 (hemagglutinin) of the H3N2 strain taken from the uniprot database http://www.uniprot.org/uniprot/P03440. These are strings of 566 characters (amino acids) and each character is replaced by the corresponding values of side-chain polarity, side-chain charge, hydropathy index and weight to obtain the feature matrix. The ground truth cluster structure is obtained based on place. The dataset https://www.kaggle.com/sandipan99/protein-dataset/ has been made public.

**Stocks**: We consider stock market dataset (the same used in [35]), where each *x*_*i*_ is a time series of daily returns for the *M* = 4000 most actively traded assets in the New York Stock Exchange, over a period from 1 January 1990 to 30 April 1999 (i.e. *d* = 2358). Returns are defined as the logarithm of the ratio between close and opening price for each day (we refer to [35] for more details). The ground truth is given by the Security and Exchange Commission (SEC) classification of the stocks in industrial sectors, that assigns a code to each stock. Taking the first two digits of the SEC code yields *S*_*σ*_ = 68 clusters (but we also compared our results with the classification based on three digits *S*_*σ*_ = 302).

**Crime**: The crime dataset https://archive.ics.uci.edu/ml/datasets/Communities+and+Crime+Unnormalized combines socio-economic data from the ‘90 Census, (law enforcement data from the 1990 Law Enforcement Management and Admin Stats survey), and crime data from the 1995 FBI UCR [36]. Typically this is a regression dataset and we bin the data points based on the values of the attributes to obtain the ground-truth cluster structure. In specific we consider three attributes which are—(i) murders per 100*k* population, (ii) robberies per 100*k* population and (iii) auto-thefts per 100*k* population.

**MNIST**: The MNIST dataset [37] http://yann.lecun.com/exdb/mnist/ consists of images of 70,000 handwritten digits (0-9). Each image is represented as a 28 × 28 pixel bounding box which we flatten to obtain a feature vector of size 784. The dataset consists of 10 classes each corresponding to a digit between 0 and 9.

## 5 Evaluation methodology

In this section we discuss in detail the evaluation methodology used in the paper.

To reiterate, we consider:

**High dimensional datasets** These are composed of *M* points $x\in R^{d}$ in a high dimensional feature space (*d* ≫ 1). For example, in stock markets data, the *i*^th^ component $x_{i}^{\left(t\right)}$ of the *i*^th^ point is the daily return of stock *i* on day *t* = 1, …, *d*.

Table 1 lists the datasets used in this study (details provided later in this section). Each consist of a set of points *x*_*i*_ *i* = 1, …, *M*. We consider different partitioning algorithms *x*_*i*_ → *s*_*i*_ that associate to each point *i* = 1, …, *M* in the sample a label *s*_*i*_ that indicates the partition to which point *x*_*i*_ belongs to. If there are *S* partitions, *s*_*i*_ can be taken as an integer between 1 and *S*.

**Table 1:** Quantitative description of data sets: *M* is the number of points, *H*[*σ*] is the entropy of the ground truth classification. *d*_1_—*d*_2_ represents the conformity among the different goodness metrics (purity, NMI and ARI) in terms of Kendall’s $\bar{\tau }$ and Spearman’s $\bar{\rho }$ rank correlation (see text). The last column reports the Kendall’s *τ* and Spearman’s *ρ* rank correlations of $\hat{H}\left[s\right]$ with the majority ranking of similarity to the ground truth (see text).

Dataset	*M*	*S*_*σ*_	$\frac{H[\sigma ]}{\text{log}\;M}$ ($\frac{H[\sigma ]}{\text{log}\;S_{\sigma }}$)	*d*_1_-*d*_2_ $\left(\bar{\tau },\bar{\rho }\right)$	*H*[*S*]-Majority (*τ*, *ρ*)
**Abalone**	4174	28	0.34 (0.85)	(0.38, 0.51)	(0.65, 0.81)
**Football**	115	12	0.52 (0.98)	(0.85, 0.93)	(0.62, 0.82)
**Railway**	301	20	0.47 (0.89)	(0.81, 0.92)	(0.89, 0.97)
**Red wine**	1598	6	0.16 (0.66)	(0.55, 0.73)	(0.56, 0.74)
**White wine**	4898	7	0.15 (0.65)	(0.48, 0.55)	(0.53, 0.73)
**Leaf**	1600	100	0.62 (1.00)	(0.78,0.90)	(0.76, 0.88)
**TREC**	878	10	0.28 (0.82)	(0.69, 0.82)	(0.52, 0.65)
**Synthetic**	1000	68	0.45 (0.74)	(0.80, 0.88)	(0.70, 0.87)
**Protein**	734	83	0.47 (0.70)	(0.66, 0.76)	(0.82, 0.93)
**Stocks (2 digits)**	4000	68	0.42 (0.82)	(0.72, 0.85)	(0.79, 0.90)
**Stocks (3 digits)**	4000	302	0.56 (0.81)	(0.85, 0.92)	(0.91, 0.96)
**Crime (murder)**	2215	45	0.30 (0.61)	(0.27, 0.31)	(0.75, 0.90)
**Crime (robbery)**	2215	46	0.33 (0.66)	(0.237, 0.34)	(0.85, 0.95)
**Crime (auto)**	2215	65	0.41 (0.76)	(0.29, 0.37)	(0.78, 0.89)
**MNIST**	70000	10	0.18 (0.90)	(0.91, 0.96)	(0.82, 0.94)

For each dataset studied, a ground truth classification *σ* = (*σ*_1_, …, *σ*_*M*_) is also available. This associates to each point *i* a “true” classification *σ*_*i*_, which can take one of *S*_*σ*_ values, where *S*_*σ*_ is the number of classes of the ground truth. For example, *σ* is the Security and Exchange Commission classification of stocks into economic sectors for financial data, or the state where a station is located for the data set of Indian railways [29]. Recall, that the classification *s* generated by a given partitioning method can be compared with the ground truth *σ*, using three well-established metrics: Purity, Normalized Mutual Information (NMI) and Adjusted Rand Index (ARI). We also compare with two existing internal metrics Davies-Bouldin (DB) and Silhouette (SH). Moreover, for the hierarchical methods, the number of clusters are set to be same as the partitional approaches. For a given data set and a given *S*, we rank algorithms according to their similarity with the ground truth.

### 5.1 Majority ranking

It is well-known that all the three similarity measures i.e., Purity, NMI and ARI have their own shortcomings [38]. This manifests in the fact that, for different similarity measures, the ranking over algorithms does not necessarily coincide. For this reason, we consider also a “majority ranking”: For algorithms *A*_1_ and *A*_2_, majority ranks *A*_1_ higher than *A*_2_ (i.e. *A*_1_ > *A*_2_) if the majority of the three similarity measures rank *A*_1_ higher than *A*_2_. This procedure is not guaranteed to produce a transitive ranking across algorithms, since it can happen that *A*_1_ > *A*_2_, *A*_2_ > *A*_3_ and *A*_3_ > *A*_1_ for some *A*_1_, *A*_2_ and *A*_3_. This signals the fact that a proper ranking is ill defined in these cases, hence we restrict attention to cases where this is not the case. As Table 1 further shows, our study covers a diverse variety of datasets, ranging from cases where the number of clusters in the ground truth is very small compared to the number of data points (red and white wines, TREC), to cases where clusters on average contain few points (football, railway). We also compare our results across different ground truths for the same dataset. For stocks we consider different levels of granularity given by the SEC codes at 2 or 3 digits. For the crime dataset we consider ground truths based on different indicators (geographic location of the community, incidence of different crimes in that community). We report the results for each case in the following subsections. The cluster size distribution also varies substantially across the data-sets used. As a measure of concentration, Table 1 reports the ratio $\hat{H}\left[\sigma \right]/\text{log}\left(S_{\sigma }\right)$ between the entropy of the cluster size distribution and its maximal value. This is one for equally sized clusters (e.g. Leaf, TREC) whereas smaller values indicate more skewed distributions.

## 6 Results

The rest of the paper will be devoted to testing the accuracy of this prediction, by comparing it with the ranking provided by the distance to the ground truth, according to the measures discussed above. We classify the datasets based on the associated ground truth cluster structure. This is to show that our metric is indeed independent of the ground truth structure. We report in detail the methodology for the stock dataset which covers the case of different granularity levels of ground truth while for other cases we mainly report the results obtained. For all these cases the same methodology has been employed to obtain the results. For general information about each dataset (size, number of clusters in the ground truth) refer to Table 1.

### 6.1 Ground truth with different granularity

**Dataset**: To illustrate, we consider stock market dataset consisting of 4000 data points and two sets of ground truth (*S*_*σ*_ = 68, 302).

**Fig 2:**
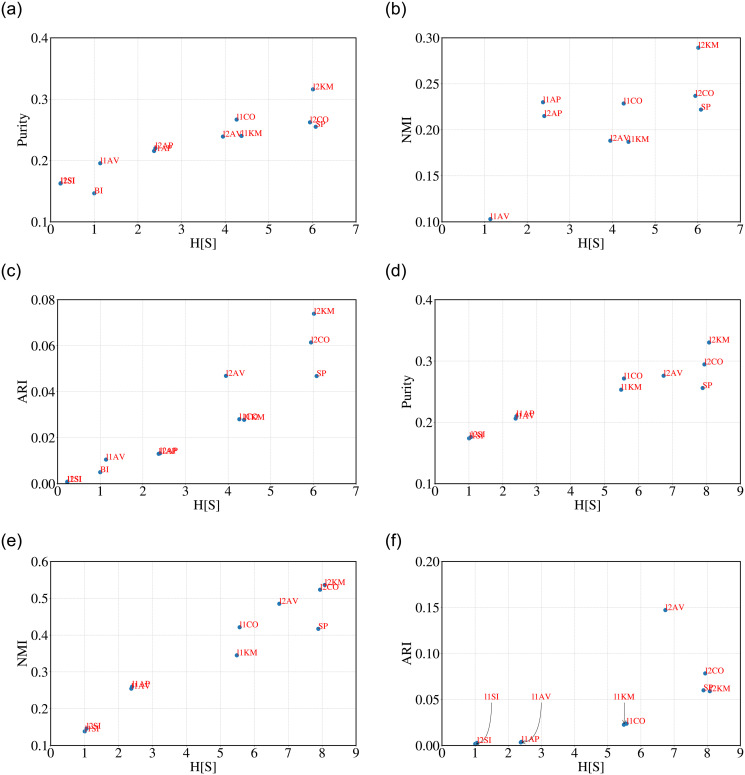
H[S] versus purity, NMI and ARI for the stock dataset, using SEC codes at 2 (top) and 3 (bottom) digits. Different algorithms are represented by a code that depends on the distance metric used (“l1” or “l2”) and the algorithm (SI, AV and CO for single, average and complete linkage, KM for k-means, AP for affinity propagation).

**Observations**: For each algorithm and choice of the measure, we compute the value of $\hat{H}\left[s\right]$ for the cluster structure obtained for *S*_*σ*_ clusters and compare it to the distance to the ground truth classification with two digits, for ARI, NMI and Purity. The plots for NMI and ARI versus $\hat{H}\left[s\right]$ in Fig 2 show a clear positive correlation that we quantify by computing the Kendall’s-*τ* and Spearman’s rank correlation *ρ* between the corresponding rankings. A pairwise comparison between $\hat{H}\left[s\right]$ and the different measures, and among the different measures, is shown in Table 2 for the stock dataset considering SEC codes at 2 digits. The corresponding results considering SEC codes at 3 digits are presented in Table 3. Different distances rank the algorithms differently and their correlation, though positive, is not one. For this reason, as already discussed, we also extract a majority ranking that combines the predictions of ARI, NMI and Purity. The correlation between majority ranking and the other rankings is also reported in Table 2 (last column). The top entry of the rightmost column (boxed) is reported in the last column of Table 1 for all the other datasets. This shows that $\hat{H}\left[s\right]$ correlates remarkably well with the majority ranking in most cases. As a comparison, we look into how the three similarity measures correlate among themselves. To this aim we calculate mean Kendall’s and Spearman’s correlation between the rankings obtained through Purity-NMI, Purity-ARI and NMI-ARI (underlined entries in Table 2). Further note that $\hat{H}\left[s\right]$ outperforms both SH and DB.

**Table 2:** Kendall’s Tau and Spearman correlation for stock considering SEC codes at 2 digits. The correlation between the majority ranking and $\hat{H}\left[s\right]$ ranking (top-right boxed entry) is reported in the last column of Table 1, whereas the average of the correlations between rankings provided by the different measures (underlined entries) is reported in the *d*_1_-*d*_2_ column of Table 1 for all datasets.

	$\hat{H}\left[s\right]$	Purity	NMI	ARI	SH	DB	Majority
$\hat{H}\left[s\right]$	1.0,1.0	0.79,0.90	0.58,0.76	0.82,0.91	-0.03,-0.08	0.45,0.60	0.79,0.90
Purity	0.79,90	1.0,1.0	0.73,0.85	0.79,0.90	-0.12,-0.22	0.48,0.65	0.94,0.98
NMI	0.58,0.76	0.73,0.85	1.0,1.0	0.64,0.81	-0.21,-0.41	0.7,0.81	0.79,0.87
ARI	0.82,0.91	0.79,0.90	0.64,0.81	1.0,1.0	-0.09,-0.14	0.64,0.76	0.85,0.95
SH	-0.03,-0.08	-0.12,-0.22	-0.21,-0.41	-0.09,-0.14	1.0,1.0	-0.15,-0.30	-0.12,-0.22
DB	0.45,0.60	0.48,0.65	0.7,0.81	0.64,0.76	-0.15,-0.30	1.0,1.0	0.55,0.69
Majority	0.79,0.90	0.94,0.98	0.79,0.87	0.85,0.95	-0.12,-0.22	0.55,0.69	1.0,1.0

**Table 3:** Kendall’s *τ* and Spearman’s correlation result for stock considering SEC codes at 3 digits.

	$\hat{H}\left[s\right]$	Purity	NMI	ARI	SH	DB	Majority
$\hat{H}\left[s\right]$	1.0,1.0	0.91,0.96	0.91,0.96	0.78,0.89	-0.07,-0.06	0.82,0.93	0.91,0.96
Purity	0.91,0.96	1.0,1.0	1.0,1.0	0.78,0.89	0.02,-0.006	0.91,0.97	1.0,1.0
NMI	0.91,0.96	1.0,1.0	1.0,1.0	0.78,0.89	0.02,-0.006	0.91,0.97	1.0,1.0
ARI	0.78,0.89	0.78,0.89	0.78,0.89	1.0,1.0	0.16,0.22	0.78,0.90	0.78,0.89
SH	-0.07,-0.06	0.02,-0.006	0.02,-0.006	0.16,0.22	1.0,1.0	0.02,0.03	0.02,-0.006
DB	0.82,0.93	0.91,0.97	0.91,0.97	0.78,0.90	0.02,0.03	1.0,1.0	0.91,0.97
Majority	0.91,0.96	1.0,1.0	1.0,1.0	0.78,0.89	0.02,-0.006	0.91,0.97	1.0,1.0

### 6.2 Ground truth built from different attributes

**Dataset**: We illustrate with the crime dataset with ground truth constructed from three attributes which are—(i) murders per 100*k* population, (ii) robberies per 100*k* population and (iii) auto-thefts per 100*k* population.

**Fig 3:**
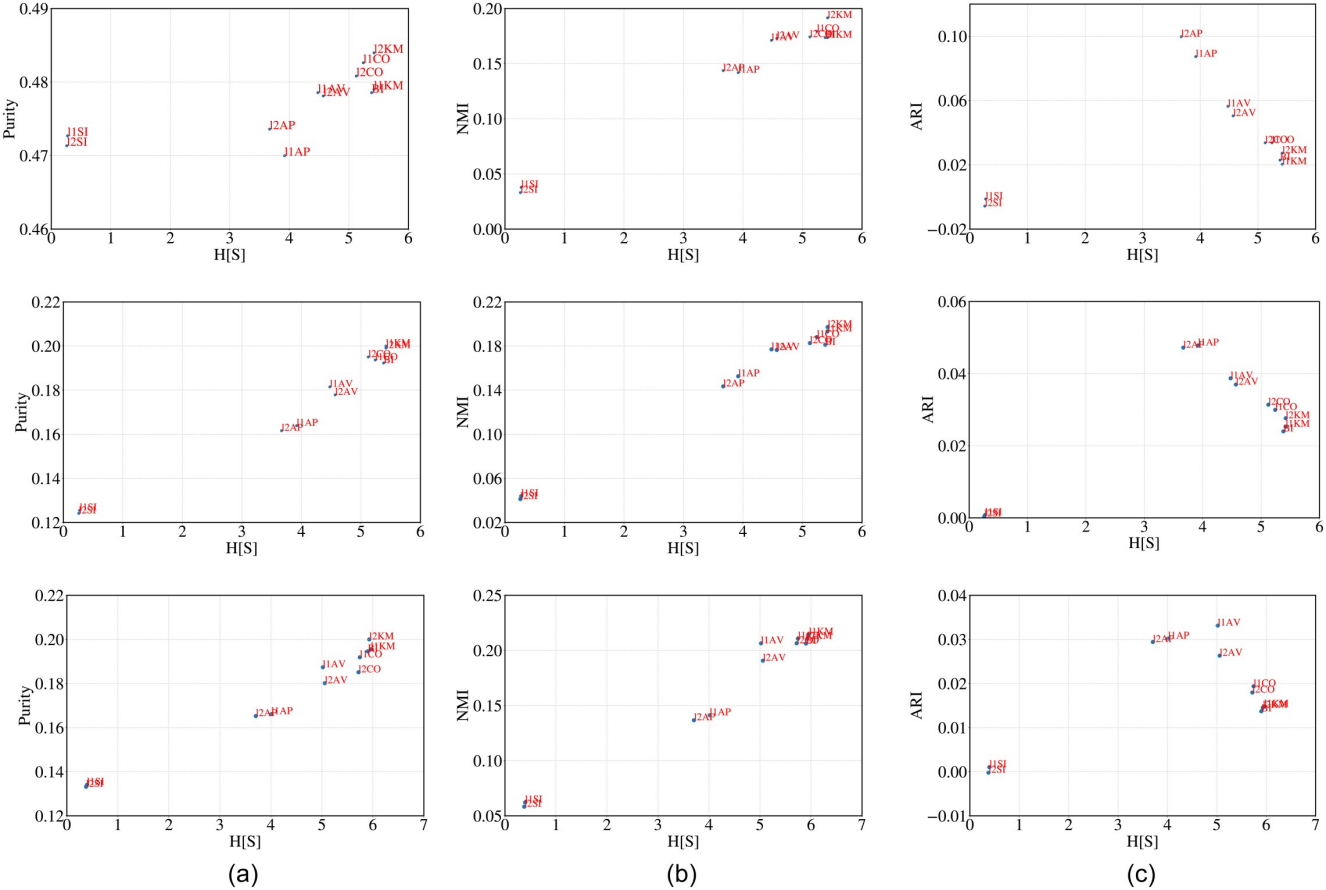
H[S] versus purity, NMI and ARI for (i) crime murder (top), (ii) crime robbery (middle) and (iii) crime auto (bottom).

**Observations**: In Fig 3(top), Fig 3(middle) and Fig 3(bottom) we plot $\hat{H}\left[s\right]$ against purity, NMI and ARI for the cluster structure obtained from each algorithm for crime murder, crime robbery and crime auto respectively. The similarity between the rankings obtained through $\hat{H}\left[s\right]$, purity, NMI, ARI and majority for the corresponding ground truths are reported in Tables 4, 5 and 6 respectively. In almost all the cases $\hat{H}\left[s\right]$ correlates highly with purity and NMI while with ARI the correlation is low. The similarity of $\hat{H}\left[s\right]$ ranking with majority is high irrespective of the ground truth used. $\hat{H}\left[s\right]$ seems to perform better than SH and DB.

**Table 4:** Kendall’s *τ* and Spearman’s correlation result for Crime (murder).

	$\hat{H}\left[s\right]$	Purity	NMI	ARI	SH	DB	Majority
$\hat{H}\left[s\right]$	1.0,1.0	0.67,0.82	0.78,0.90	-0.2,-0.05	0.82,0.94	0.56,0.79	0.75,0.90
Purity	0.67,0.82	1.0,1.0	0.82,0.92	-0.02,0.03	0.56,0.75	0.6,0.72	0.85,0.94
NMI	0.78,0.91	0.82,0.92	1.0,1.0	0.02,0.07	0.67,0.83	0.71,0.84	0.96,0.99
ARI	-0.2,-0.05	-0.02,0.03	0.02,0.07	1.0,1.0	-0.16,-0.03	0.09,0.10	0.05,0.08
SH	0.82,0.94	0.56,0.75	0.67,0.83	-0.16,-0.03	1.0,1.0	0.38,0.62	0.64,0.81
DB	0.56,0.79	0.6,0.72	0.71,0.84	0.09,0.10	0.38,0.62	1.0,1.0	0.75,0.85
Majority	0.75,0.90	0.85,0.94	0.96,0.99	0.05,0.08	0.64,0.80	0.75,0.85	1.0,1.0

**Table 5:** Kendall’s *τ* and Spearman’s correlation result for Crime (robbery).

	$\hat{H}\left[s\right]$	Purity	NMI	ARI	SH	DB	Majority
$\hat{H}\left[s\right]$	1.0,1.0	0.82,0.94	0.89,0.96	-0.16,-0.04	0.82,0.94	0.56,0.79	0.85,0.95
Purity	0.82,0.94	1.0,1.0	0.93,0.98	-0.05,0.02	0.71,0.87	0.67,0.82	0.96,0.99
NMI	0.89,0.96	0.93,0.98	1.0,1.0	-0.05,0.02	0.71,0.87	0.67,0.82	0.96,0.99
ARI	-0.16,-0.04	-0.05,0.02	-0.05,0.02	1.0,1.0	-0.13,-0.02	0.05,0.11	-0.02,0.03
SH	0.82,0.94	0.71,0.87	0.71,0.87	-0.13,-0.02	1.0,1.0	0.38,0.62	0.75,0.81
DB	0.56,0.79	0.67,0.82	0.67,0.82	0.05,0.11	0.38,0.62	1.0,1.0	0.64,0.81
Majority	0.85,0.95	0.96,0.99	0.96,0.99	-0.02,0.03	0.75,0.81	0.64,0.81	1.0,1.0

**Table 6:** Kendall’s *τ* and Spearman’s correlation result for Crime (auto).

	$\hat{H}\left[s\right]$	Purity	NMI	ARI	SH	DB	Majority
$\hat{H}\left[s\right]$	1.0,1.0	0.89,0.96	0.82,0.91	-0.05,-0.009	0.78,0.90	0.64,0.81	0.78,0.89
Purity	0.89,0.96	1.0,1.0	0.78,0.90	-0.02,0.05	0.82,0.92	0.6,0.76	0.82,0.91
NMI	0.82,0.91	0.78,0.90	1.0,1.0	0.13,0.18	0.60,0.76	0.75,0.88	0.96,0.99
ARI	-0.05,-0.009	-0.02,0.05	0.13,0.18	1.0,1.0	0.02,0.08	0.16,0.15	0.16,0.22
SH	0.78,0.91	0.82,0.92	0.60,0.76	0.02,0.08	1.0,1.0	0.42,0.57	0.64,0.77
DB	0.64,0.81	0.60,0.76	0.75,0.88	0.16,1.15	0.42,0.57	1.0,1.0	0.71,0.83
Majority	0.78,0.89	0.82,0.91	0.96,0.99	0.16,0.22	0.64,0.77	0.71,0.83	1.0,1.0

### 6.3 Small number of ground truth clusters compared to the number of points

**Datasets**: For this scenario, we consider wine and TREC datasets here. For TREC *M* = 878 and *S*_*σ*_ = 10 and the corresponding numbers for red and white wines are *M* = 1598, *S*_*σ*_ = 6 and *M* = 4598, *S*_*σ*_ = 7 respectively. MNIST consists of 70000 data points and 10 clusters (i.e., *M* = 70000 and *σ* = 10).

**Fig 4:**
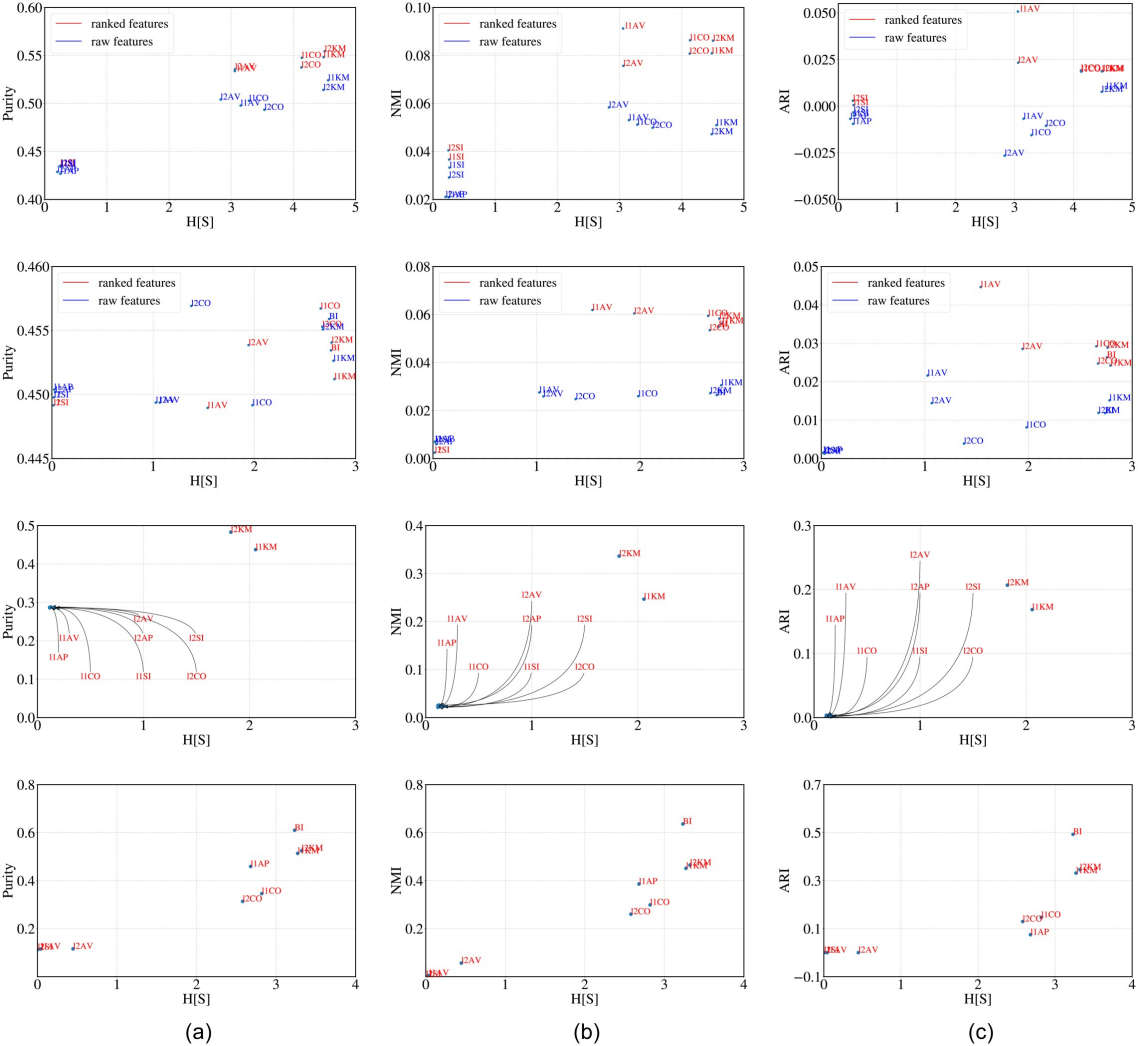
H[S] versus purity, NMI and ARI for (i) red wine, (ii) white wine, (iii) TREC and (iv) MNIST datasets (from top to bottom). Note that for the wine datasets we considered two types of feature matrices. For raw features (represented in blue) we considered the values of the features as provided in the dataset to obtain the feature vector of each point while for ‘ranked feature” (represented in red) we rank each feature based on the value and then use this rank score instead of the raw value.

**Observations**: We plot $\hat{H}\left[s\right]$ against purity, NMI and ARI for the cluster structure obtained from each algorithm for red wine (top), white wine, TREC and MNIST (bottom) in Fig 4 (top to bottom in the same order). The similarity scores between the rankings obtained through $\hat{H}\left[s\right]$, purity, NMI, ARI and majority are reported in Tables 7 and 8 for the respective wine datasets. In both these cases rankings obtained through $\hat{H}\left[S\right]$, correlates only moderately with the majority ranking. In fact, the similarity values are low among the rankings obtained through other metrics as well. The similarity is reasonably high for TREC (refer to Table 9) and MNIST (refer to Table 10).

**Table 7:** Kendall’s *τ* and Spearman’s correlation result for Red Wine.

	$\hat{H}\left[s\right]$	Purity	NMI	ARI	SH	DB	Majority
$\hat{H}\left[s\right]$	1.0,1.0	0.66,0.82	0.45,0.64	0.23,0.42	0.06,0.08	0.32,0.42	0.56,0.74
Purity	0.66,0.82	1.0,1.0	0.76,0.91	0.46,0.69	-0.12,-0.12	0.45,0.49	0.90,0.96
NMI	0.45,0.64	0.76,0.91	1.0,1.0	0.44,0.60	-0.15,-0.13	0.35,0.42	0.87,0.95
ARI	0.23,0.42	0.46,0.69	0.44,0.60	1.0,1.0	-0.24,-0.46	0.33,0.48	0.57,0.73
SH	0.06,0.08	-0.12,-0.12	-0.15,-0.13	-0.24,-0.46	1.0,1.0	0.11,0.24	-0.18,-0.14
DB	0.32,0.42	0.45,0.49	0.35,0.42	0.33,0.48	0.11,0.24	1.0,1.0	0.42,0.48
Majority	0.56,0.74	0.90,0.96	0.87,0.95	0.57,0.73	-0.18,-0.14	0.42,0.48	1.0,1.0

**Table 8:** Kendall’s *τ* and Spearman’s correlation result for White Wine.

	$\hat{H}\left[s\right]$	Purity	NMI	ARI	SH	DB	Majority
$\hat{H}\left[s\right]$	1.0,1.0	0.31,0.54	0.56,0.73	0.52,0.69	-0.15,-0.18	0.41,0.55	0.53,0.73
Purity	0.31,0.54	1.0,1.0	0.26,0.35	0.28,0.33	-0.11,-0.11	0.24,0.42	0.29,0.37
NMI	0.56,0.73	0.26,0.35	1.0,1.0	0.92,0.98	-0.21,-0.21	0.26,0.33	0.93,0.98
ARI	0.52,0.69	0.28,0.33	0.92,0.98	1.0,1.0	-0.19,-0.19	0.26,0.32	0.95,0.99
SH	-0.15,-0.18	-0.11,-0.11	-0.21,-0.21	-0.19,-0.19	1.0,1.0	-0.40,-0.50	-0.22,-0.22
DB	0.41,0.55	0.24,0.42	0.26,0.33	0.26,0.32	-0.40,-0.49	1.0,1.0	0.27,0.35
Majority	0.53,0.73	0.29,0.37	0.93,0.98	0.95,0.99	-0.22,-0.22	0.27,0.35	1.0,1.0

**Table 9:** Kendall’s *τ* and Spearman’s correlation result for TREC.

	$\hat{H}\left[s\right]$	Purity	NMI	ARI	SH	DB	Majority
$\hat{H}\left[s\right]$	1.0,1.0	0.33,0.41	0.60,0.80	0.42,0.61	-0.56,-0.71	0.78,0.89	0.52,0.65
Purity	0.33,0.41	1.0,1.0	0.63,0.76	0.64,0.79	-0.07,-0.10	0.20,0.29	0.64,0.84
NMI	0.60,0.80	0.64,0.76	1.0,1.0	0.82,0.92	-0.33,-0.53	0.47,0.72	0.82,0.93
ARI	0.42,0.61	0.66,0.79	0.82,0.92	1.0,1.0	-0.31,-0.56	0.43,0.64	0.81,0.93
SH	-0.56,-0.70	-0.07,-0.10	-0.33,-0.53	-0.33,-0.56	1.0,1.0	-0.78,-0.92	-0.33,-0.45
DB	0.78,0.89	0.20,0.29	0.47,0.72	0.43,0.64	-0.78,-0.92	1.0,1.0	0.38,0.58
Majority	0.52,0.65	0.64,0.84	0.82,0.93	0.81,0.93	-0.33,-0.45	0.38,0.58	1.0,1.0

**Table 10:** Kendall’s *τ* and Spearman’s correlation result for MNIST.

	$\hat{H}\left[s\right]$	Purity	NMI	ARI	SH	DB	Majority
$\hat{H}\left[s\right]$	1.0,1.0	0.87,0.95	0.87,0.95	0.82,0.94	0.16,0.15	0.47,0.68	0.82,0.94
Purity	0.87,0.95	1.0,1.0	1.0,1.0	0.87,0.95	0.11,0.09	0.51,0.67	0.96,0.98
NMI	0.87,0.95	1.0,1.0	1.0,1.0	0.87,0.95	0.11,0.09	0.51,0.67	0.96,0.98
ARI	0.82,0.94	0.87,0.95	0.87,0.95	1.0,1.0	-0.02,-0.03	0.64,0.79	0.91,0.96
SH	0.16,0.15	0.11,0.09	0.11,0.09	-0.02,-0.03	1.0,1.0	-0.2,-0.23	0.07,0.04
DB	0.47,0.68	0.51,0.67	0.51,0.67	0.64,0.79	-0.2,-0.23	1.0,1.0	0.56,0.68
Majority	0.82,0.94	0.96,0.98	0.96,0.98	0.91,0.96	0.07,0.04	0.56,0.68	1.0,1.0

### 6.4 Ground truth clusters with very few points

**Datasets**: We consider the examples of football (*M* = 115, *S*_*σ*_ = 12) and railway (*M* = 301, *S*_*σ*_ = 20) datasets.

**Fig 5:**
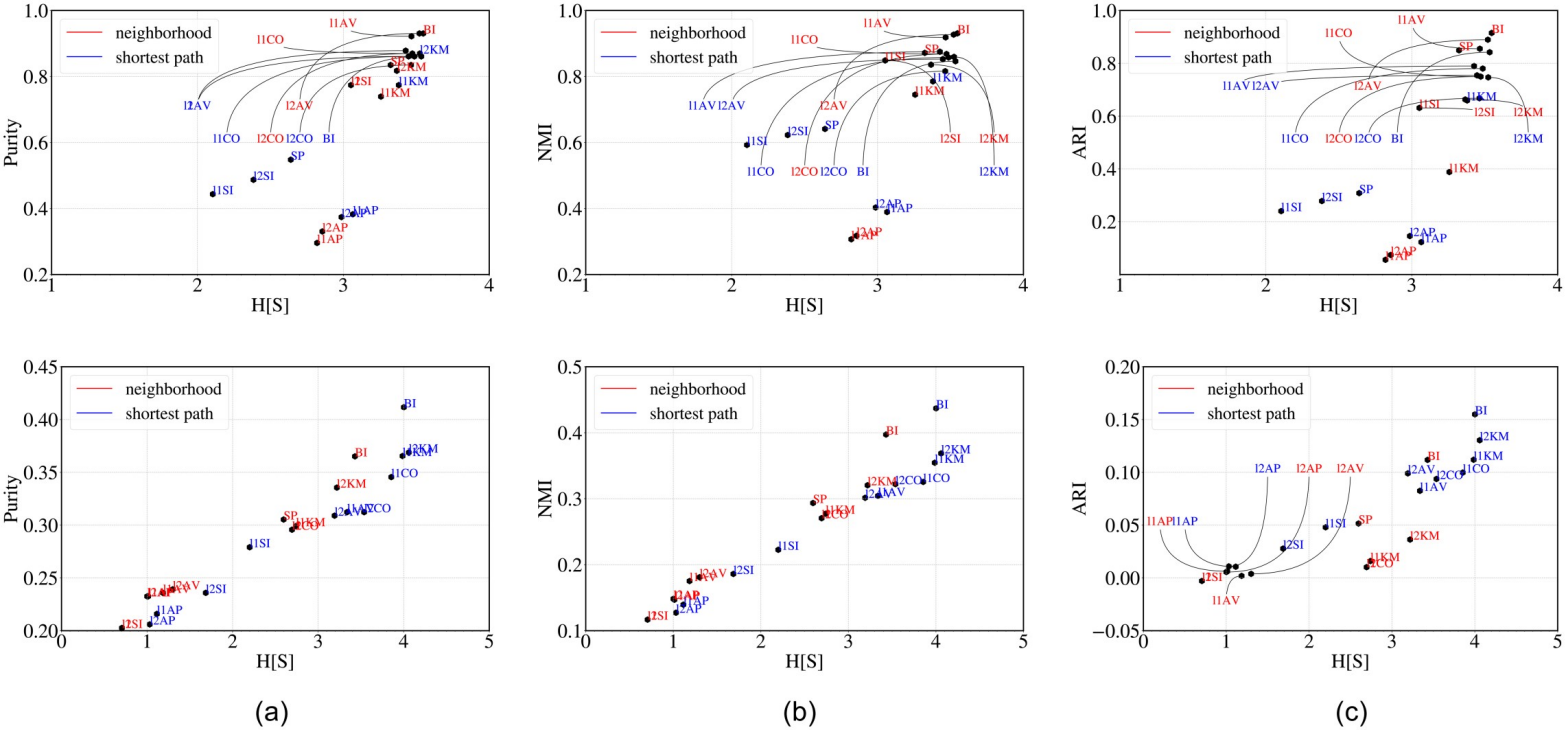
H[S] versus purity, NMI and ARI for (i) football (top) and (ii) railway (bottom). We consider two types of feature vectors for each data point (node). In case of ‘neighborhood” (represented in blue) the feature vector of each node *u*_*i*_ consists of 1s and 0s depending on whether *u*_*j*_(*j* ≠ *i*) is a neighbor or not. For ‘shortest path” (represented in red) the feature vector of each node ui consists of the shortest path to *u*_*j*_(*j* ≠ *i*).

**Observations**: In Fig 5 (top) and (bottom) we plot $\hat{H}\left[s\right]$ against purity, NMI and ARI for the cluster structure obtained from each algorithm for football and railway. $\hat{H}\left[s\right]$ is indeed closely related with the other metrics in both cases which proves the effectiveness our metric. We further report the similarity among various rankings of the clustering algorithms obtained through the different metrics in Tables 11 and 12. In fact we observe a very high correlation between $\hat{H}\left[s\right]$ and majority ranking.

**Table 11:** Kendall’s *τ* and Spearman’s correlation result for Football.

	$\hat{H}\left[s\right]$	Purity	NMI	ARI	SH	DB	Majority
$\hat{H}\left[s\right]$	1.0,1.0	0.68,0.87	0.57,0.75	0.65,0.83	0.6,0.79	-0.01,-0.015	0.62,0.82
Purity	0.68,0.87	1.0,1.0	0.87,0.94	0.84,0.93	0.78,0.88	0.22,0.34	0.89,0.96
NMI	0.57,0.75	0.87,0.94	1.0,1.0	0.84,0.93	0.73,0.87	0.34,0.45	0.92,0.97
ARI	0.65,0.83	0.84,0.93	0.84,0.93	1.0.,1.0	0.75,0.87	0.30,0.39	0.89,0.96
SH	0.6,0.79	0.78,0.88	0.73,0.87	0.75,0.87	1.0,1.0	0.16,0.25	0.77,0.90
DB	-0.01,-0.015	0.22,0.34	0.34,0.45	0.30,0.39	0.16,0.25	1.0,1.0	0.26,0.39
Majority	0.62,0.82	0.89,0.96	0.92,0.97	0.89,0.96	0.77,0.90	0.26,0.39	1.0,1.0

**Table 12:** Kendall’s *τ* and Spearman’s correlation result for Railway.

	$\hat{H}\left[s\right]$	Purity	NMI	ARI	SH	DB	Majority
$\hat{H}\left[s\right]$	1.0,1.0	0.88,0.97	0.89,0.97	0.76,0.89	0.49,0.66	0.39,0.55	0.89,0.97
Purity	0.88,0.97	1.0,1.0	0.94,0.99	0.74,0.88	0.47,0.64	0.46,0.61	0.94,0.99
NMI	0.89,0.97	0.94,0.99	1.0,1.0	0.75,0.90	0.45,0.63	0.45,0.61	0.97,0.99
ARI	0.76,0.89	0.74,0.88	0.75,0.90	1.0,1.0	0.4,0.54	0.36,0.46	0.75,0.90
SH	0.49,0.66	0.47,0.64	0.45,0.63	0.4,0.54	1.0,1.0	-0.05,-0.07	0.45,0.64
DB	0.39,0.55	0.46,0.61	0.45,0.61	0.36,0.46	-0.05,-0.07	1.0,1.0	0.42,0.60
Majority	0.89,0.97	0.94,0.99	0.97,0.99	0.75,0.90	0.45,0.64	0.42,0.60	1.0,1.0

### 6.5 Ground truth clusters are of equal sizes

**Datasets**: Here we consider the leaf and the abalone datasets. While for leaf the number of points in each ground truth cluster is exactly 16, the corresponding number for abalone is ∼ 90.

**Fig 6:**
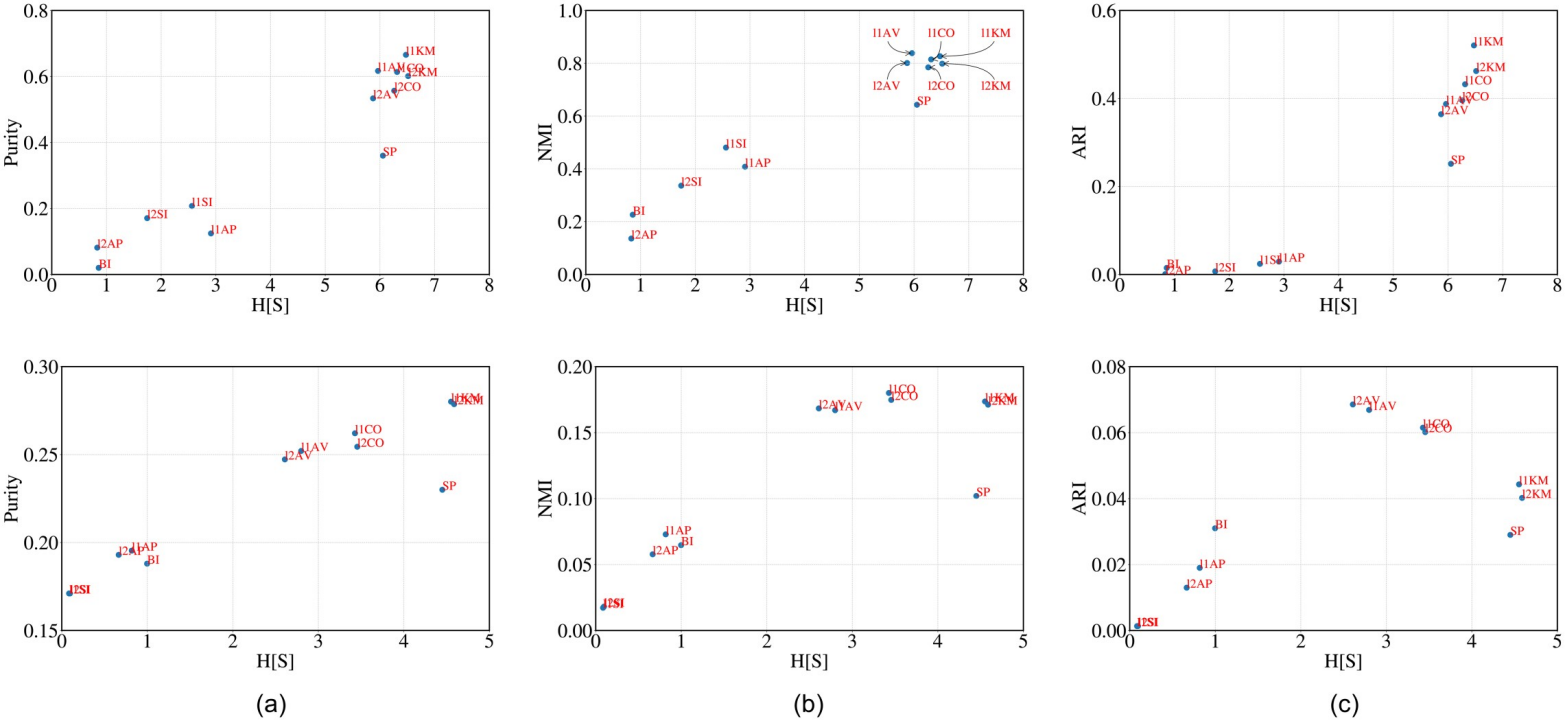
H[S] versus purity, NMI and ARI for Leaf (top) and Abalone (below) datasets.

**Observations**: In Fig 6(top) and (bottom) we plot $\hat{H}\left[s\right]$ against purity, NMI and ARI values of the cluster structure obtained as output from all the clustering algorithms. A strong positive dependence suggests that $\hat{H}\left[s\right]$ is able to correctly rank the performance of the clustering algorithms. High correlation between the rankings of clustering algorithms obtained through $\hat{H}\left[s\right]$ and majority (refer to Tables 13 (leaf) and 14 (abalone)) further supports our hypothesis.

**Table 13:** Kendall’s *τ* and Spearman’s correlation result for Leaf.

	$\hat{H}\left[s\right]$	Purity	NMI	ARI	SH	DB	Majority
$\hat{H}\left[s\right]$	1.0,1.0	0.70,0.86	0.67,0.79	0.88,0.96	0.52,0.71	0.55,0.57	0.76,0.88
Purity	0.7,0.86	1.0,1.0	0.85,0.95	0.76,0.90	0.52,0.74	0.36,0.36	0.94,0.98
NMI	0.67,0.79	0.85,0.95	1.0,1.0	0.73,0.86	0.61,0.81	0.33,0.38	0.91,0.97
ARI	0.88,0.96	0.76,0.90	0.73,0.86	1.0,1.0	0.58,0.78	0.55,0.55	0.82,0.93
SH	0.52,0.71	0.52,0.74	0.61,0.81	0.58,0.78	1.0,1.0	0.24,0.44	0.58,0.78
DB	0.55,0.56	0.36,0.36	0.33,0.38	0.55,0.55	0.24,0.44	1.0,1.0	0.42,0.44
Majority	0.76,0.88	0.94,0.98	0.91,0.97	0.82,0.93	0.58,0.78	0.42,0.44	1.0,1.0

**Table 14:** Kendall’s *τ* and Spearman’s correlation result for Abalone.

	$\hat{H}\left[s\right]$	Purity	NMI	ARI	SH	DB	Majority
$\hat{H}\left[s\right]$	1.0,1.0	0.73,0.88	0.64,0.81	0.39,0.59	0.15,0.34	0.61,0.80	0.64,0.81
Purity	0.73,0.88	1.0,1.0	0.79,0.92	0.48, 0.72	0.18,0.41	0.58,0.73	0.79,0.92
NMI	0.64,0.81	0.79,0.92	1.0,1.0	0.7,0.81	0.33,0.51	0.42,0.63	1.0,1.0
ARI	0.39,0.59	0.48,0.72	0.7,0.81	1.0,1.0	0.64,0.80	0.12,0.24	0.7,0.81
SH	0.15,0.34	0.18,0.41	0.33,0.51	0.64,0.80	1.0,1.0	-0.18,-0.13	0.33,0.51
DB	0.61,0.80	0.58,0.73	0.42,0.63	0.12,0.24	-0.18,-0.13	1.0,1.0	0.42,0.63
Majority	0.64,0.81	0.79,0.92	1.0,1.0	0.7,0.81	0.33,0.51	0.42,0.63	1.0,1.0

### 6.6 Ground truth cluster sizes are skewed

**Datasets**: Here we consider the synthetic and the protein datasets where the ground truth cluster size distributions are skewed.

**Fig 7:**
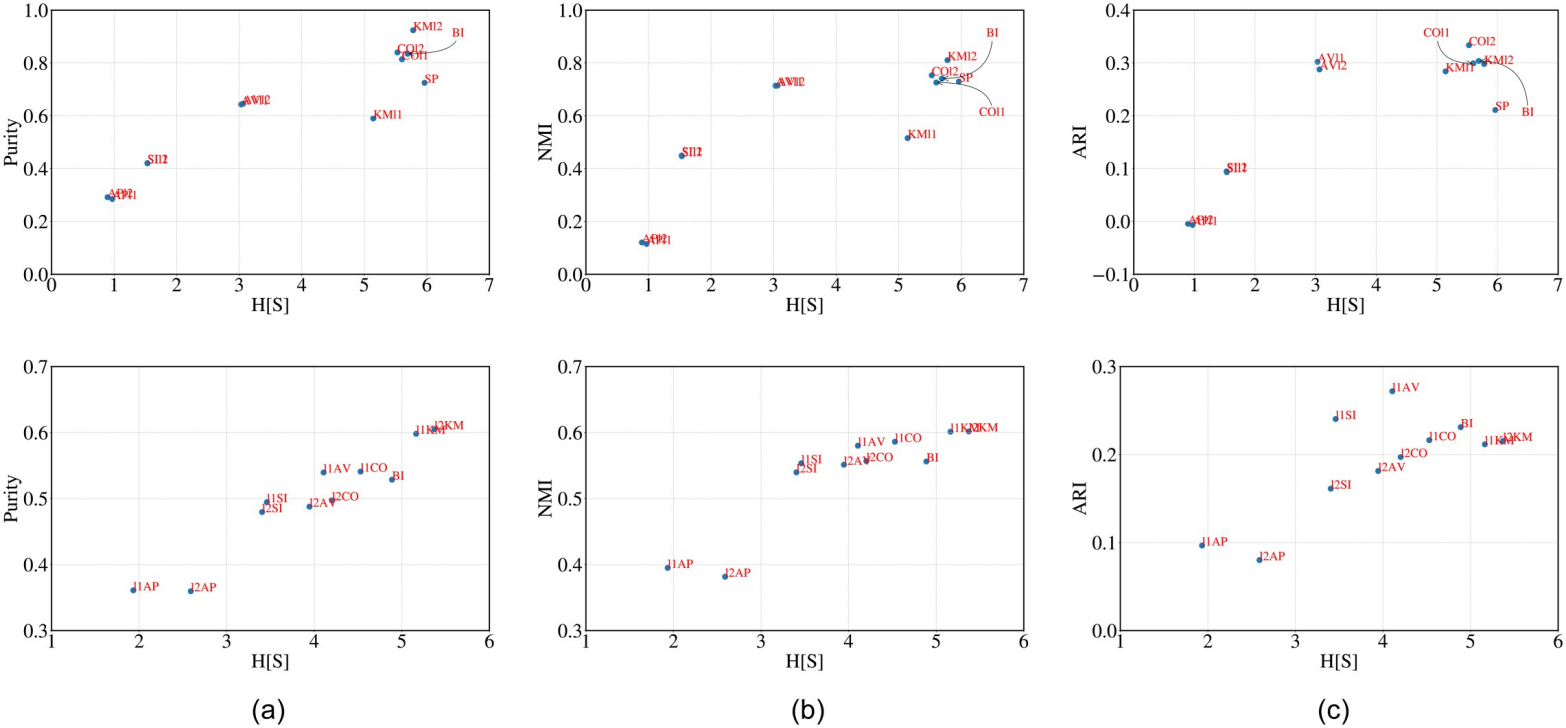
H[S] versus purity, NMI and ARI for Synthetic (top) and Protein (below) datasets.

**Observations**: It can be clearly observed from the Fig 7 top (synthetic) and bottom (protein) that $\hat{H}\left[s\right]$ correlates nicely with other metrics in measuring the goodness of the cluster structure obtained as output from different clustering algorithms. Higher similarity (refer to Table 15 (synthetic) and Table 16 (protein)) between the majority ranking and that obtained through $\hat{H}\left[s\right]$ further indicates the effectiveness of our metric in ranking the performance of the clustering algorithms.

**Table 15:** Kendall’s *τ* and Spearman’s correlation result for Synthetic.

	$\hat{H}\left[s\right]$	Purity	NMI	ARI	SH	DB	Majority
$\hat{H}\left[s\right]$	1.0,1.0	0.70,0.87	0.73,0.89	0.42,0.62	0.48,0.58	0.67, 0.83	0.70,0.87
Purity	0.70,0.87	1.0,1.0	0.97,0.99	0.73,0.84	0.73,0.84	0.36,0.62	1.0,1.0
NMI	0.73,0.89	0.97,0.99	1.0,1.0	0.7,0.81	0.7,0.81	0.39,0.64	0.97,0.99
ARI	0.42,0.62	0.73,0.84	0.69,0.81	1.0,1.0	0.71,0.86	0.27,0.51	0.73,0.84
SH	0.48,0.58	0.73,0.84	0.7,0.81	0.70,0.84	1.0,1.0	0.21,0.28	0.73,0.85
DB	0.67,0.83	0.36,0.62	0.39,0.64	0.27,0.51	0.21,0.28	1.0,1.0	0.36,0.62
Majority	0.70,0.87	1.0,1.0	0.97,0.99	0.73,0.84	0.73,0.85	0.36,0.62	1.0,1.0

**Table 16:** Kendall’s *τ* and Spearman’s correlation result for Protein.

	$\hat{H}\left[s\right]$	Purity	NMI	ARI	SH	DB	Majority
$\hat{H}\left[s\right]$	1.0,1.0	0.82,0.93	0.78,0.91	0.42,0.54	0.13,0.14	0.27,0.31	0.82,0.93
Purity	0.82,0.93	1.0,1.0	0.96,0.99	0.53,0.67	0.02,0.018	0.24,0.26	1.0,1.0
NMI	0.78,0.91	0.96,0.99	1.0,1.0	0.49,0.63	-0.02,-0.01	0.20,0.25	0.96,0.99
ARI	0.42,0.54	0.53,0.67	0.49,0.63	1.0,1.0	0.13,0.09	-0.09,-0.145	0.53,0.67
SH	0.13,0.14	0.02,0.02	-0.02,-0.01	0.13,0.09	1.0,1.0	0.42,0.54	0.02,0.02
DB	0.27,0.31	0.24,0.26	0.2,0.25	-0.09,-0.14	0.42,0.54	1.0,1.0	0.24,0.26
Majority	0.82,0.93	1.0,1.0	0.96,0.99	0.53,0.67	0.02,0.02	0.24,0.26	1.0,1.0

### 6.7 Summary

To summarize we showed that performance of $\hat{H}\left[s\right]$ is comparable to the other metrics even though it does not require the ground truth cluster structure unlike the other competing metrics. Through extensive experiments on a large variety of datasets we showed that our proposed metric is indeed effective as well as robust. This further indicate that $\hat{H}\left[s\right]$ is independent of the associated ground truth structure. $\hat{H}\left[s\right]$ also consistently outperforms both the baseline internal metrics across all the datasets.

### 6.8 Dependence on cluster structure

We have demonstrated that the proposed metric is able to outperform the existing internal metrics across different datasets. We now focus on analysing dependence of the performance of our metric on the complexity of the dataset. To quantify the complexity of a dataset we define two metrics $q_{1}=\frac{\hat{H}\left[\sigma \right]}{\text{log}\;M}$ and $q_{2}=\frac{H[\sigma ]}{\text{log}\;S_{\sigma }}$ where $\hat{H}\left[\sigma \right]$ measures the entropy of the ground truth cluster for the dataset. For *q*_1_, $\hat{H}\left[\sigma \right]$ is normalized by the number of points in the dataset (log *M* in specific) while for *q*_2_ it is normalized by the number of clusters in the ground truth (log *S*_*σ*_). Note that we calculate these two metrics for each dataset (refer to Table 1 for exact values) and train a linear regression model to predict the performance ($\hat{H}\left[S\right]-Majority\left(\tau ,\rho \right)$) on each dataset. We obtain a reasonably high *R*^2^ of 0.52. This indicates that complexity of the dataset in terms of *q*_1_ and *q*_2_ is indeed correlated to the performance of the proposed metric.

## 7 Discussion

The results discussed in this paper suggest that Infomax can be used as a completely unsupervised measure, that can be computed solely from the partition size distribution, for each algorithm. Using this, we can rank data clustering algorithms in an unsupervised manner.

**On community detection**. A closely related problem, that of community detection in networks, has received considerable attention recently in Physics. The core idea is to group nodes in the network based on structural similarity. As in case of clustering, there exists a plethora of algorithms for community detection as well. An immediate extension would be to deploy our proposed metric to the problem of ranking community detection algorithms.

On experimenting with various datasets we observed that

1. The performance of clustering algorithms depends on the dataset. In case of the football dataset we observed that average linkage was performing the best whereas in case of the railway dataset *k*-means was performing the best.
2. The performance of clustering algorithms also depends on the distance metric used for calculating distance between the data points in the dataset. This dependence is different depending on the algorithm. For example, in the crime dataset, l2 distance performs better than l1 in *k*-means, but worse than l1 in complete linkage.
3. The performance changes depending on the feature matrix used.

These observations reinforces the conclusion [8] that the search for the perfect clustering algorithm is chimeric. This makes it important to develop unsupervised methods to rank partitioning algorithms as the one we presented in this paper.
